# Editorial: Mediterranean foods: quality, safety and sustainability

**DOI:** 10.3389/fnut.2024.1370508

**Published:** 2024-02-06

**Authors:** Ana Cristina Agulheiro-Santos, Marta Laranjo, Carmen Jarén

**Affiliations:** ^1^MED-Mediterranean Institute for Agriculture, Environment and Development and CHANGE-Global Change and Sustainability Institute, IIFA-Institute for Advanced Studies and Research, Universidade de Évora, Évora, Portugal; ^2^Departamento de Fitotecnia, Escola de Ciências e Tecnologia, Universidade de Évora, Évora, Portugal; ^3^Departamento de Medicina Veterinária, Escola de Ciências e Tecnologia, Universidade de Évora, Évora, Portugal; ^4^Departamento de Ingeniería, Universidad Pública de Navarra, Pamplona, Spain; ^5^Institute for Sustainability and Food Chain Innovation—ISFOOD, Pamplona, Spain

**Keywords:** Mediterranean diet, sustainability, food safety, food quality, sustainable development goals

In recent years, the Mediterranean diet has been recovered, especially after its recognition as UNESCO's intangible cultural heritage. It involves the use of many plant-based foods common to several Mediterranean countries, such as olive oil, olives, fruits and vegetables, cereals, pulses, nuts, wine, but also meat and fish. The adoption of this diet has favorable and direct implications on health, but also on society and economy, with consequences for the sustainability and resilience of agrifood systems, inherent to production, relevant topics in the current context of climate change and water scarcity.

Additionally, these Research Topics are aligned with the 2030 Agenda of the United Nations, mainly contributing to Sustainable Development Goals 2 (Zero Hunger), 3 (Good Health and Wellbeing), and 12 (Responsible Consumption). In this twenty-first century, new challenges have been imposed on all of us involving the food distribution chain, from producers to consumers, including researchers. In parallel with food security, the access to safe food, and the reduction of food loss and waste are also urgent challenges to be addressed.

To achieve these worldwide objectives, it is necessary to explore innovative strategies for production of raw materials, to transform unexploited into new food raw materials, to use new manufacturing processes, as well as innovative conservation methods. All these objectives contribute to the availability and accessibility of quality foods that enable an increased adherence to the Mediterranean diet and should be achieved taking environmental concerns into account.

The Research Topic on “*Mediterranean foods: quality, safety and sustainability*” focuses on different Mediterranean diet foods, including their relationship with environmental sustainability and production systems.

Among the submitted manuscripts, four research articles were selected by external experts to enter this Research Topic of Frontiers in Nutrition.

The work by Scarano et al. deals with the phytochemical characterization of strawberry tree (*Arbutus unedo* L.) fruit extracts. The authors focused on the chemical profile of bioactive compounds at different ripening stages.

In the study by López-Maestresalas et al., the authors present a method to classify potatoes according to their crispness aptitude after cooking using near-infrared hyperspectral imaging (NIRS).

Tang et al. studied a dragon-kiwi fermented beverage and discovered that this functional fruit fermented beverage has a good antioxidant activity both *in vitro* and *in vivo*. Moreover, this antioxidant capacity is stable during an *in vitro* simulated digestion process in in *Caenorhabditis elegans*.

Finally, Bankole et al. evaluated the rheological and nutraceutical benefits of common types of yogurts and their production processes, including the incorporation of natural and modified additives into yogurt.

Furthermore, the authors of the four articles come from four different countries, two European (Italy and Spain) and two non-European, namely China and Nigeria.

We are happy to launch this Research Topic, which includes four manuscripts that reported new findings on fermented foods. We hope that the readers of *Frontiers in Nutrition* find this *Mediterranean foods: quality, safety and sustainability* Research Topic of relevance to their research area.

## Author contributions

AA-S: Writing—original draft, Writing—review & editing. ML: Writing—original draft, Writing—review & editing. CJ: Writing—original draft, Writing—review & editing.

